# Central Texas Medical Association

**Published:** 1900-07

**Authors:** 


### The Medical Societies.

### Central Texas Medical Association.

The Thirteenth Semi-Annual Session of the Central Texas Medical Association will convene in Waco, Texas, Tuesday and Wednesday, July 10 and 11, 1900. The enclosed program contains many subjects of importance and those who have agreed to furnish papers are men of known ability and experience.

You are full of practical ideas, picked up along the pathway of life, and we are anxious to have the benefit of your co-operation.

He cannot count it as time lost, who has spent a few days in acquiring knowledge of men and things pertaining to his profession. If a member of the society, it is your duty to attend its meetings and participate in its council. If not a member, then you can make no mistake by coming out and joining during the July meeting.

Yours fraternally,
A. C. SCOTT, President.

N. A. OLIVE, Secretary.

TUESDAY, JULY 10, 1900.—FORENOON SESSION, 10 A. M.

*Section of Obstetrics and Gynaecology:*

A. R. Kuykendall, M. D., Chairman; J. H. Alexander, M. D., Secretary.

1. Report of Chairman.
2. "Preparation of Lying-in Patient and Management of Normal Labor." Paper by J. H. Alexander, M. D., Meridian. Discussion by Drs. M. B. Grace and C. C. Foster.
3. "Causes of Abnormal and Difficult Labor and their Treatment." Paper by J. A. Winfrey, Hico. Discussion by Drs. R. B. Sellers and J. E. Brown.
4. "When to Repair Injuries of Cervix and Perineum in the Parturient, and the Operative Technic." Paper by Dr. Hoppel, Cleburne. Discussion by Drs. Albert L. Taylor and J. C. Woods.
5. "The Causes, Sequellæ and Treatment of Pelvic Peritonitis in the Female." Paper by E. B. Osborne, M. D., Cleburne. Discussion by Drs. R. W. Park and O. I. Halbert.
6. "Cause and Treatment of Acute and Chronic Endometritis." Paper by R. L. Kimmins, M. D., Iredell. Discussion by Drs. C. J. Crow and C. L. Clay.

JULY 10, 1900.—AFTERNOON SESSION, 2 P. M.

*Section of Nervous Diseases and Medical Jurisprudence:*

John H. Turner, M. D., Chairman; M. L. Graves, M. D., Secretary.

1. "Neurasthenia." Paper by E. D. Bondurant, M. D., Mobile, Ala. Discussion by Drs. H. C. Ghent and M. L. Graves.
2. "Hysteria." Paper by Dr. Tutweiler, Flatonia. Discussion by Drs. W. L. Blailock and J. H. Sears.
3. "The Education and Proper Care of the Feeble Minded." Paper by C. T. Wilbur, M. D., Kalamazoo, Mich. Discussion by Drs. D. R. Wallace and B. E. Hadra.
4. "Cerebral Traumatism." Paper by Frank R. Ross, M. D., Austin. Discussion by Drs. J. W. Hale and C. E. Smith.
5. "Neurasthenia." Paper by J. W. Lea, M. D. Discussion by Drs. A. H. Snead and Frank R. Ross.
6. "The Insane from the Standpoint of the General Practitioner." Paper by G. H. Moody, M. D., San Antonio. Discussion by Drs. J. W. Lea and A. M. Curtis.
7. "Training of Weak Minded Children." Paper by Jno. P.

Stewart, M. D., Farmdale, Ky. Discussion by Drs. G. H. Moody and W. C. Blailock.

8. "Eleemosynary Jurisprudence." Paper by Jno. S. Turner, M. D., Terrell. Discussion by Drs. D. R. Wallace and M. L. Graves.

EVENING SESSION, 8:30 P. M., JULY 10, 1900.

1. "The Legal Aspect of Quarantine." Paper by Capt. H. C. Lindsey, Waco. Discussion by Felix D. Robinson, Esq., and Dr. O. I. Halbert.

WEDNESDAY, JULY 11, 1900.—FORENOON SESSION, 10 A. M.

*Section of Ophthalmology and Otology:*

Dr. J. M. Woodson, Chairman.

1. "Atrophic Rhinitis." Paper by Dr. J. M. Woodson. Discussion by Drs. J. R. Ferrel and W. F. Cole.

2. "Neurotic Mastoidities." Paper by Dr. Jno. O. McReynolds. Discussion by Drs. W. B. Anderson and J. W. Hale.

*Section of General Medicine:*

J. W. Embree, M. D., Chairman; W. R. Blailock, Secretary.

1. "Medical Treatment of Appendicitis." Paper by E. C. Gordon, M. D., Lott. Discussion by Drs. W. W. Greer and C. K. Haggard.

2. "Scarlet Fever." Paper by W. H. Nixon, M. D., Killeen. DIscussion by Drs. J. B. Young and P. M. Kuykendall.

3. "Feeding of Infants." Paper by J. F. Haley, M. D., Oenaville. Discussion by Drs. J. M. Johnson and W. R. McCormack.

4. "Case of Heart Disease." Paper by J. F. Siln, M. D., New York. Discussion by Drs. W. O. Wilkes and Daniel Parker.

5. "Serum Therapy." Paper by J. S. McCelvey, M. D., Temple. Discussion by Drs. J. H. Sears and W. C. Blailock.

6. "Hypnotism." Paper by J. E. Seay, M. D., Pratt City, Ala. Discussion by Drs. H. C. Black and Walter Wilcox.

7. "Hysteria." Paper by M. P. McElhannon, M. D., Belton. Discussion by Drs. R. P. Talley and H. W. Brown.

8. "Then 1885—Now 1900." Paper by H. C. Ghent, M. D. Discussion by Drs. J. C. J. King and A. H. Snead.

AFTERNOON SESSION, 2 P. M., JULY 11, 1900.

*Section of Surgery:*

W. C. Blailock, M. D., Chairman; J. S. McCelvey, M. D., Secretary.

1. "Treatment of Wounds by the General Practitioner." Paper by A. M. Curtis, M. D., Waco. Discussion by Drs. J. D. Law and J. R. Gillam.

2. "Surgical Treatment of Appendicitis." Paper by Taylor Hudson, M. D., Belton. Discussion by Drs. E. C. Gordon and G. B. Foscue.

3. Subject Announced. Paper by W. R. Blailock, M. D.

4. "Appendicitis." Paper by J. W. Hale, M. D. Discussion by Drs. M. M. Edmondson and J. B. Shelmire.

5. "Calpo-Hysterectomy." Paper by R. H. Simpson, M. D., Turnersville. Discussion by Drs. S. L. Terrell and W. T. Baird.

6. "Appendicitis." Paper by J. R. Stuart, M. D., Houston. Discussion by Drs. A. H. Snead and H. C. Black.

---

